# Chromatin status and transcription factor binding to gonadotropin promoters in gonadotrope cell lines

**DOI:** 10.1186/s12958-017-0304-z

**Published:** 2017-10-24

**Authors:** Huimin Xie, Hanne M. Hoffmann, Anita K. Iyer, Melissa J. Brayman, Cindy Ngo, Mary Jean Sunshine, Pamela L. Mellon

**Affiliations:** 1 Department of Reproductive Medicine, Center for Reproductive Science and Medicine, University of California, 9500 Gilman Drive La Jolla, San Diego, CA 92093-0674 USA; 2 Illumina Inc, 5200 Illumina Way, San Diego, CA 92122 USA; 3 Foley and Lardner LLP, 402 West Broadway, Suite 2100, San Diego, CA 92101 USA

**Keywords:** Epigenetic, DNA accessibility, Histone modification, Gonadotrope development, ChIP Assay, Chromatin

## Abstract

**Background:**

Proper expression of key reproductive hormones from gonadotrope cells of the pituitary is required for pubertal onset and reproduction. To further our understanding of the molecular events taking place during embryonic development, leading to expression of the glycoproteins luteinizing hormone (LH) and follicle-stimulating hormone (FSH), we characterized chromatin structure changes, imparted mainly by histone modifications, in model gonadotrope cell lines.

**Methods:**

We evaluated chromatin status and gene expression profiles by chromatin immunoprecipitation assays, DNase sensitivity assay, and RNA sequencing in three developmentally staged gonadotrope cell lines, αT1–1 (progenitor, expressing *Cga*), αT3–1 (immature, expressing *Cga* and *Gnrhr*), and LβT2 (mature, expressing *Cga*, *Gnrhr*, *Lhb*, and *Fshb*), to assess changes in chromatin status and transcription factor access of gonadotrope-specific genes.

**Results:**

We found the common mRNA α-subunit of LH and FSH, called *Cga*, to have an open chromatin conformation in all three cell lines. In contrast, chromatin status of *Gnrhr* is open only in αT3–1 and LβT2 cells. *Lhb* begins to open in LβT2 cells and was further opened by activin treatment. Histone H3 modifications associated with active chromatin were high on *Gnrhr* in αT3–1 and LβT2, and *Lhb* in LβT2 cells, while H3 modifications associated with repressed chromatin were low on *Gnrhr*, *Lhb,* and *Fshb* in LβT2 cells. Finally, chromatin status correlates with the progressive access of LHX3 to *Cga* and *Gnrhr*, followed by PITX1 binding to the *Lhb* promoter.

**Conclusion:**

Our data show the gonadotrope-specific genes *Cga*, *Gnrhr*, *Lhb*, and *Fshb* are not only controlled by developmental transcription factors, but also by epigenetic mechanisms that include the modulation of chromatin structure, and histone modifications.

## Background

The pituitary arises from the closure of Rathke’s pouch on mouse embryonic day 12 (E12.5), giving rise to five endocrine cell types: gonadotropes, lactotropes, corticotropes, thyrotropes, and somatotropes. The development of these specialized cells depends on a stringent temporal and spatial control of transcription factors [1, 2]. The gonadotropes emerge late in development at ~E16.5, and comprise ~10–20% of pituitary cells in adulthood. The major role of gonadotropes is to regulate puberty and fertility through the synthesis and secretion of luteinizing hormone (LH) and follicle-stimulating hormone (FSH). FSH and LH are dimeric glycoproteins, formed from an α and a β subunit. The β subunit is distinct for each hormone, and is transcribed from separate genes. Gonadotrope cells can be traced throughout pituitary development by the sequential appearance of early lineage markers. First the mRNA for the common α-subunit of LH and FSH (*Cga*, glycoprotein hormones also known as αGSU) is detectable at E11.5 in the mouse, followed by the nuclear receptor Steroidogenic Factor 1 (*Sf1* or *Nr5a1*), and the gonadotropin-releasing hormone receptor (*Gnrhr*). Finally, the gonadotropin hormone beta subunit mRNAs emerge at E16.5 for *Lhb* and E17.5 for *Fshb* [3].

Specification of cell fate is controlled by a combination of transcription factors acting on cis-regulatory elements, as well as epigenetic mechanisms that include the modulation of chromatin structure [4, 5]. Gene activation and repression are specifically regulated through changes in chromatin structure imparted mainly by histone modifications and DNA methylation. Inactive genes typically display condensed chromatin that is resistant to DNaseI digestion, and show histone H3 deacetylation and methylation [6, 7]. Active genes and regulatory elements are often in an open chromatin conformation to facilitate binding of regulatory proteins.

The access of transcription factors to the chromatin requires the relaxation of chromatin, leading to an open conformation and increased sensitivity to DNAseI digestion [8–10]. The regulation of chromatin status is complex and its analysis requires multiple complementary approaches. Open chromatin is often associated with acetylation at histone H3 and tri-methylation at histone H3-lysine 4 (H3K4) [6, 7]. In addition, certain combinations of histone modifications are thought to distinguish different regions within a given gene, i.e., promoters versus enhancers. For example, active promoters show high levels of tri-methylation and low levels of mono-methylation at H3K4; whereas enhancers show the opposite methylation pattern at H3K4 [11]. The dynamic nature of chromatine modifications, are key in they role to control chromatine compaction and gene expression. Indeed, histone deacetylases (HDAC) allow histone deacetylation, leading to chromatin compaction [12, 13]. The conversion of chromatin from an inactive to active state at genes involved in differentiation is thought to promote the maturation of progenitor and precursor cells, however the chromatin status changes taking place during gonadotrope maturation are still poorly understood due to the complex cell composition of the pituitary.

Molecular investigation of the regulation of gonadotrope gene expression has been greatly facilitated by the use of well-characterized, cultured cell lines that represent different maturation stages of gonadotropes [14–17]. The αT1–1 cell line represents a progenitor to the gonadotrope and/or thyrotrope lineages [15] and expresses the single common glycoprotein hormone subunit gene, *Cga* [18]. The immature gonadotrope αT3–1 cell line expresses both *Cga* and *Gnrhr*, and the mature gonadotrope LβT2 cell line expresses all four gonadotrope-specific genes *Cga*, *Gnrhr*, *Lhb*, and *Fshb* [5, 15, 19, 20]. We and others have previously shown that these unique cell lines are excellent model systems for investigating the molecular mechanisms of gonadotrope differentiation [5, 15, 19, 21–26]. Moreover, these cell lines express the known tissue-specific regulators of the four gonadotrope-specific differentiated target genes including *Sf1, Lhx3, Pitx1, Runx, Foxl2*, and *Gata2* [22, 24, 25, 27–31], and their binding sites in the proximal promoters of the gonadotrope-specific genes have been defined either experimentally or bioinformatically [5, 29, 32–34]. These tissue-specific transcription factors play direct roles in regulating the transcription of the gonadotrope-specific target genes, yet the coordinated program of gonadotrope maturation remains to be elucidated [35, 36]. Recent work has begun to address this topic by analyzing the epigenetic regulation of gonadotrope specific genes [5]. To further our understanding of the global chromatin status of the gonadotrope-specific genes during gonadotrope maturation, and obtain a more comprehensive understanding of the changes in chromatin status allowing specific expression of key gonadotrope markers, we investigated chromatin status and its correlation to gonadotrope gene expression in our model pituitary lineage cell lines.

## Methods

### Cell culture

To establish how chromatin status on key gonadotrope genes changes during gonadotrope cell maturation, we studied three model immortalized mouse gonadotrope cell lines αT1–1, αT3–1, and LβT2, as well as two control cell lines, the mouse thyrotrope cell line TαT1, and the mouse fibroblast cell line, NIH3T3 (ATCC). All cell lines were cultured in DMEM with 4.5% glucose (Mediatech Inc., Herndon, VA), 10% fetal bovine serum (Gemini Bio, West Sacramento, CA), and 1× penicillin-streptomycin (Life Technologies, Inc./Invitrogen, Grand Island, NY) in 5% CO_2_ at 37 °C. Cells were seeded on 10 cm dishes (Nunc, Roskilde, Denmark) and harvested at subconfluency. For hormone treatment of LβT2 cells, cells were serum-starved for 16 h in DMEM containing 4.5% glucose, 1× penicillin-streptomycin and 0.1% bovine serum albumin. Cells were then treated for 4 h with 100 ng/ml GnRH (Sigma-Aldrich, St. Louis, MO) ± 25 ng/ml activin (Calbiochem, La Jolla, CA) before they were harvested for the DNase sensitivity assay.

### DNase sensitivity assay

Actively transcribed chromatin is characterized by being in an open conformation, allowing easy access of transcription factors to their binding sites. This open chromatin increases the sensitivity of the chromatin to DNaseI. To establish to what degree the gonadotrope promoters of model gonadotrope cell lines would increase their sensitivity to DNaseI treatment during maturation we performed a DNase sensitivity assay. DNase sensitivity assay was performed as previously described [37]. In brief, αT1–1, αT3–1, LβT2, and NIH3T3 cells were lysed in hypotonic buffer and nuclei were isolated by centrifugation at 2200 g for 5 min at 4 °C. Intact nuclei were resuspended in 1X DNaseI Reaction Buffer (Promega, Madison, WI) containing 2% glycerol. Equal amounts of nuclei were added to increasing quantities of DNaseI (Promega) in 1X DNase Reaction Buffer, ranging from 0 units (U) to 7.5 U, and incubated at 37 °C for 5 min. DNaseI was inactivated using DNaseI Stop Solution (Promega) and incubation at 65 °C for 10 min. Treated nuclei were lysed in Nuclei Lysis Buffer followed by RNase A and Proteinase K digestion. Genomic DNA was then isolated by extraction twice with phenol/chloroform/isoamyl alcohol and once with chloroform. DNA was ethanol precipitated and resuspended in Tris-EDTA buffer followed by 55 °C incubation for one hour. qPCR was performed using SYBR Green supermix and an iQ5 real-time PCR machine (BioRad). Primer sequences are detailed in Table 1. Forty ng of DNA from each treatment condition was quantitated relative to a standard curve of dilutions of undigested DNA. Data from each primer set were normalized to the active gene, *Actb*, or to the vehicle treatment as stated in the figure legends [37].

**Table 1:** Primers used for qRT-PCR for ChIP and DNase sensitivity assays

Primer		Product length	Chrom-osome	Localization	
Name	Sequence (5′-3′)			Start	End
Actb-F	GGCCAGCGTTTGCCTTTTATGGTAATAAT	181	5	142,905,689	142,905,869
Actb-R CGAACTATCAAGACACAAAAGAAGGCTATA					
Fshb-F	GGTGTGCTGCCATATCAGATTCGG	280	2	107,059,594	107,059,873
Fshb-R GCATCAAGTGCTGCTACTCACCTGTG					
Gnrh-F	CAGCAGGTGTTGCAATTACATTCACCATTAAG	227	14	67,745,100	67,745,326
Gnrh-R CCTGTTTGGATGTGAAAGTCAAAGGGATCTC					
Cga-F	GAAAATGGCCAAATGCTCTC	193	4	34,893,533	34,893,725
Cga-R TGTTCCCAGCTGCACATAAG					
Lhb-F	CGAGTGTGAGGCCAATTCACTGG	218	7	45,420,767	45,420,984
Lhb-R GGGCCCTACCATCTTACCTGGAGC					
Gnrhr-F	ATCAGAAGTAACAGGGACTCCACTC	202	5	86,197,672	86,197,873
Gnrhr-R AGGCAGTAGAGAGTAGGAAAAGGAAG					

Localization and product length (bp) from the UCSC Genome Browser using the Mouse Dec. 2011 (GRCm38/mm10) Assembly.

### Chromatin immunoprecipitation (ChIP)

To evaluate transcription factor binding to key gonadotrope gene promoters, we performed ChIP assays. ChIP assays were performed as previously described [38]. Chromatin was sonicated to an average length of 300–500 bp using a Branson Sonifier 250 (Branson Ultrasonics Corp., Danbury, CT). Antibodies recognizing specific histone modifications were: anti-acetyl-Histone-H3 (06–599; Millipore, Temecula, CA), anti-trimethyl-Histone H3-Lys4 (07–473; Millipore), anti-dimethyl-Histone H3-Lys9 (ab1220; Abcam), all of which are ChIP-grade antibodies. To recognize phosphorylated polymerase, ChIP-grade anti-RNA polymerase II CTD repeat YSPTSPS (phospho S5) (ab5131; Abcam, Cambridge, MA), was used. Immunoprecipitated DNA and DNA from input chromatin were analyzed for sequences of interest by qRT-PCR using promoter-spanning primers specified in Table 1. For ChIP assays comparing αT1–1, αT3–1, and LβT2 chromatin, the percentage of enrichment of antibody signal over IgG was calculated for each primer set. IgG was the same species and source as the comparison antibody. Then, values for activating chromatin marks were normalized to those for the inactive gene *Gnrh1*. For repressive chromatin marks, values were normalized to the highly active gene *Actb* [37–39].

### Association of transcription factors by ChIP

Antibodies were ChIP grade or previously validated for ChIP assays: anti-LHX3 (L2202, US Biological, MA) [25], anti-PITX1 (sc-18,922X, Santa Cruz, ChIP grade). Immunoprecipitated DNA was analyzed for sequences of interest by qRT-PCR using promoter-specific primers shown in Table 1. For ChIP assays comparing αT1–1, αT3–1, and LβT2 chromatin, the percentage of enrichment of antibody signal over IgG was calculated for each primer set. Values were then normalized to those for the inactive gene *Gnrh1* [37, 38].

### RNA sequencing

To establish if gene expression levels in the studied cell lines correlated with chromatin status, we performed RNA sequencing. RNA was isolated from αT1–1, αT1–3, LβT2, TαT1, and NIH3T3 cells using TRIzol® (Invitrogen, Grand Island, NY), as per the manufacturer’s instructions, and treated with Turbo DNA-free DNase (Ambion, Life Technology, USA). The RNA integrity (RNA Integrity Number ≥ 9) and quantity was determined on the Agilent 2100 Bioanalyzer (Agilent, Palo Alto, CA, USA). cDNA libraries (*n* = 2) were created using the TruSeq™ RNA Sample Prep-v2 (Illumina, San Diego, CA), using the manufacturer’s low-throughput protocol. Indexed samples were mixed at equal concentrations, four samples per lane, and sequenced using the HiSeq 2000 sequencer (Illumina). The resulting sequences were aligned to the mouse genome using the Illumina Consensus Assessment of Sequence and Variation (CASAVA) software program. qRT-PCR, sequencing, and alignment were performed by the UCSD BIOGEM Core facility supported by NIH grants P30 DK063491 and P30 CA023100. Thirty-six bp, single end sequencing of the five cell lines generated an average of 3.6 GB of sequencing data per sample with the following characteristics: NIH3T3: 852.2 Mbp, 23.67 million reads and passing filter % (PF%) of 75.6; αT1–1: reads, αT3–1: 933.2 Mbp, 25.92 million reads and PF% of 75.2; LβT2: 897.0 Mbp, 24.92 million reads and PF% of 67.8; and TαT1: 887.4 Mbp, 24.65 million reads and PF% of 70.7.

### Statistical analysis

All experiments were repeated independently at least three times. In the figures, the error bars represent the SEM. Data were analyzed by one or two-way ANOVA in GraphPad Prism 7 (La Jolla, CA). For all analyses, the result was considered significant if *P* ≤ 0.05.

## Results

### Chromatin density on gonadotrope-specific genes during development

**Fig. 1:**
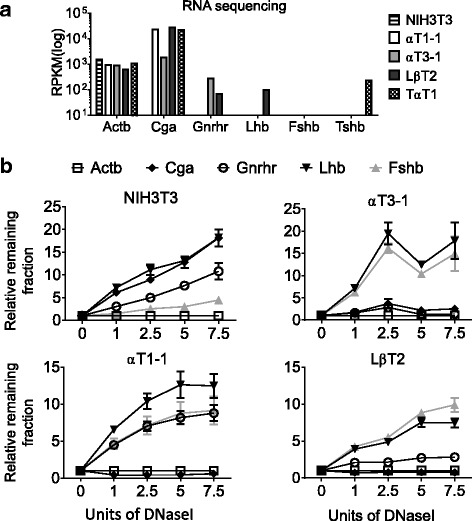
Chromatin accessibility of gonadotrope-specific genes during development. **a** Expression levels of gonadotrope genes in αT1–1, αT3–1, LβT2, TαT1, and NIH3T3 cell lines (*n* = 2). **b** DNaseI sensitivity assays in NIH3T3, αT1–1, αT3–1, and LβT2 cells. DNA from nuclei was treated with increasing concentrations of DNaseI and analyzed by qRT-PCR with primers specific to regulatory elements (Table 1). Amplicon quantities were normalized to the active *Actb* gene, and qRT-PCR data are presented as the mean fraction of DNA remaining relative to *Actb* ± SEM

To confirm that the immortalized pituitary cell lines expressed the key pituitary lineage markers and provide direct quantitation of expression levels, we performed RNA sequencing. We found that all five of the cell lines expressed high levels of β-actin (Fig. 1a, *Actb*). As expected, the common gonadotrope hormone α subunit, *Cga,* was expressed in all the gonadotrope lineage cell lines, as well as the thyrotrope cell line, TαT1 [15, 18], but not in NIH3T3 cells (Fig. 1a). The immature (αT3–1) and mature (LβT2) pituitary cell lines both expressed *Gnrhr,* whereas the gonadotrope marker, *Lhb*, was only expressed by LβT2 cells. The thyrotrope cell line TαT1 [18] was the only cell line studied expressing the thyrotrope marker *Tshb,* and did not express any of the gonadotrope-specific genes, other than the common α subunit, *Cga* (Fig. 1a). We did not detect *Fshb* in LβT2 cells, although these cells are known to express this mRNA as detected by the more sensitive method of qRT-PCR. *Fshb* is found at low levels that can be induced by activin treatment [19, 40, 41]. NIH3T3 cells were used as a negative control for pituitary gene expression (Fig. 1a).

To determine if chromatin status correlates with expression of gonadotrope genes, we investigated the chromatin status by DNaseI sensitivity assay [42]. We focused on chromatin accessibility of *Cga*, *Gnrhr*, *Lhb*, and *Fshb* in the gonadotrope lineage cell lines αT1–1, αT3–1, and LβT2, which represent different stages of gonadotrope development. NIH3T3 cells were used as a negative control. *Actb* (β-actin) is ubiquitously expressed in all the above cell lines, reflecting an open chromatin status, and was therefore used to normalize the DNaseI data (Fig. 1a and b, *Actb*). Three classes of chromatin accessibility have been determined by this assay: closed (repressed), poised, and open (active) [43, 44]. One of the characteristics of chromatin is how dynamic its conformation is, and its capacity to compact and unfold. Thus, degrees of compaction and opening of the DNA are possible. Normalizing our data to the highly transcribed *Actb* allowed us to determine the relative DNaseI sensitivity of the studied gonadotrope genes. To reveal the degree of accessibility of the DNA, we used increasing amounts of DNaseI. As expected, we found that *Cga, Lhb*, and *Gnrhr* were much less sensitive to DNaseI treatment than *Actb* (Fig. 1b), which correlated with gene expression levels (Fig. 1a). Unexpectedly, *Fshb* was sensitive to DNaseI treatment, although to a lesser extent than *Actb* (Fig. 1b). However this did not correlate with gene transcription (Fig. 1a), supporting the importance of using more than one approach to study chromatin status to obtain a complete image of chromatin accessibility to transcription factors. *Cga* is the earliest gonadotrope expressed gene (E11.5 in the mouse embryo) and has high expression in the gonadotrope progenitor αT1–1 cell line (Fig. 1a). Consistent with this, we found an open chromatin structure in the promoter region of *Cga* in this cell line (Fig. 1b, αT1–1). The chromatin states of all other later chromatin modifications of gonadotrope cell lines were repressed as evidenced by their structures being closed and resistant to DNaseI degradation (Fig. 1b, αT1–1, *Fshb, Lhb* and *Gnrhr*). The immature gonadotrope cell-line αT3–1 expresses both *Cga* and *Gnrhr* (Fig. 1a). In agreement with this, we determined the chromatin state of these genes to be open (Fig. 1b, αT3–1). *Lhb* and *Fshb* are not expressed in the αT3–1 cells and have closed chromatin in this cell (Fig. 1a). Our data show that the chromatin of *Lhb* and *Fshb* started to shift from closed to poised in the LβT2 cells (Fig. 1b, LβT2, Two-way ANOVA, followed by Sidaks multiple comparison, *p* > 0.01 at 7.5 Units of DNaseI). The only partial opening of *Lhb* and *Fshb* in LβT2 cells is not surprising, as cells in culture are not in the tridimensional environment they experience in vivo and do not receive hormonal stimulation, as gonadotropes would in vivo, allowing them to fully activate expression of *Lhb* and *Fshb*.

### Chromatin modifications of gonadotrope-specific genes during development

**Fig. 2:**
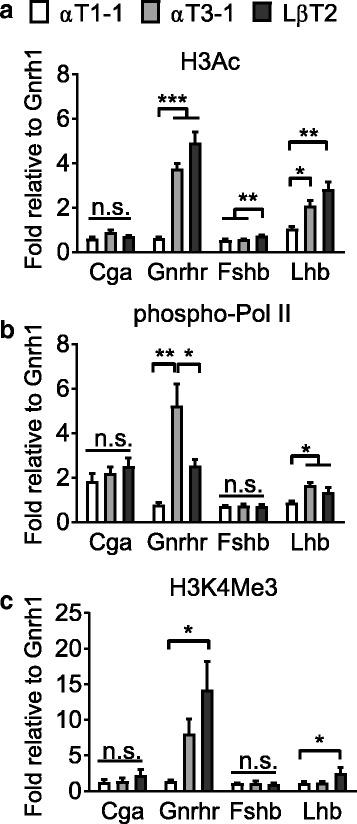
Active histone modifications and recruitment of phospho-Pol II to gonadotrope-specific genes in gonadotrope cells. ChIP analysis using antibodies specific to (**a**) H3Ac, (**b**) phospho-Pol II, and (**c**) H3K4Me3 in αT1–1, αT3–1, and LβT2 cells. Immunoprecipitated chromatin from αT1–1, αT3–1, and LβT2 cells was subjected to qRT-PCR. Data are presented as fold enrichment relative to IgG, with values normalized to *Gnrh1* and expressed as mean ± SEM (*n* = 4). Statistical analysis by one-way ANOVA followed by a Tukey post-hoc, * *p* < 0.05, ** *p* < 0.01, *** *p* < 0.001, as indicated by bar. n.s.: non-significant

Numerous modifications of chromatin and histones allow for successful recruitment of phosphorylated RNA polymerase II (phospho-Pol II), which is required to initiate transcription [10]. To determine if the opening of the chromatin in these cell lines correlates with a change in the acetylation and methylation signatures on the histones binding to the promoters and allows occupation with phospho-Pol II, we next performed ChIP assays. We used antibodies recognizing the activating histone modification marks histone H3 acetylation (H3Ac), and H3K4 trimethylation (H3K4Me3, Fig. 2), as well as recruitment of phospho-Pol II [38, 44]. Although, *Cga* is highly expressed by all three gonadotrope lineages (Fig. 1a), and the promoter is sensitive to DNaseI treatment (Fig. 1b), the active histone marks in all three cell lines were surprisingly little enriched as compared to *Gnrh1*, despite recruitment of phospho-Pol II and active transcription (Fig. 1a and 2a). This is unexpected and is possibly due to the chosen region of study of the *Cga* promoter. In agreement with both transcription levels of *Gnrhr*, and its promoter’s sensitivity to DNaseI treatment, the *Gnrhr* regulatory region went from possessing very few active histone marks in αT1–1, to highly enriched in these marks in αT3–1 and LβT2 cells (Fig. 2). In agreement with our findings using the DNaseI sensitivity assay, which found the *Fshb* promoter to be in a rather closed conformation (Fig. 1b), this promoter exhibits only one of the chromatin marks of an open promoter (H3Ac), but does not bind phosphorylated polymerase II (Fig. 2). Finally, the chromatin status of *Lhb* is enriched in H3Ac and recruits phosphorylated polymerase II to the regulatory region somewhat in αT3–1 and more strongly in LβT2, which correlated with transcription in LβT2 cells where it also exhibits enrichment of H3K4Me3 (Fig. 1a and 2).

**Fig. 3:**
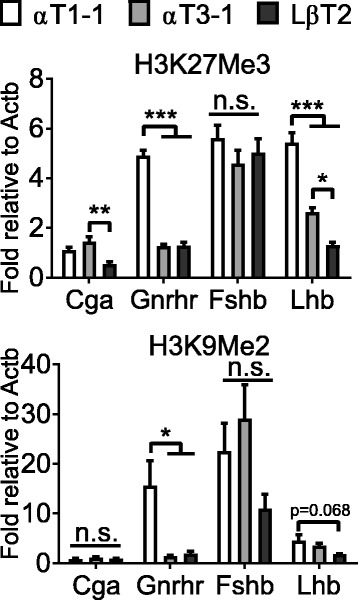
Histone modifications of inactive chromatin in αT1–1, αT3–1, and LβT2 cells. ChIP analysis was performed using antibodies specific to H3K27Me3, and H3K9Me2. Immunoprecipitated chromatin from αT1–1, αT3–1, and LβT2 cells was subjected to qRT-PCR. Data are presented as fold enrichment relative to IgG, with values normalized to *Actb* and expressed as mean ± SEM. Statistical analysis by one-way ANOVA followed by a Tukey post-hoc, * *p* < 0.05, ** *p* < 0.01, *** *p* < 0.001, as indicated by bar. n.s.: non-significant

To assess the epigenetic role of repression, we performed ChIP assays to analyze the repressive histone modifications H3K9Me2 and H3K27Me3 [45]. We found that one or both modifications were reduced with developmental maturation on the promoters of *Gnrhr* and *Lhb* (Fig. 3). *Gnrhr* loses both repressive marks in αT3–1 and LβT2 cells, while *Lhb* has progressively reduced H3K27Me3 from αT1–1 to αT3–1 and finally LβT2 and trends toward lower H3K9Me2 in LβT2 cells (Fig. 3). Whereas no changes were observed on the *Fshb* promoter (Fig. 3).

### Sequential recruitment of developmental pituitary transcription factors to gonadotrope-specific promoters

**Fig. 4:**
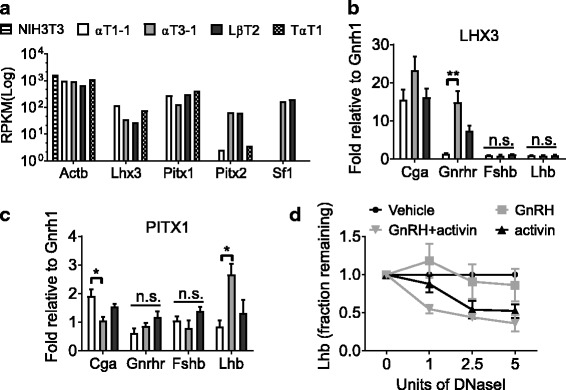
Pituitary transcription factor accessibility in developing gonadotrope cell lines. **a** Expression levels of transcription factors in NIH3T3, αT1–1, αT3–1, LβT2, and TαT1 cell lines (*n* = 2). ChIP assays in αT1–1, αT3–1, and LβT2 cells were performed using antibodies specific to the transcription factors (**b**) LHX3 and (**c**) PITX1. Immunoprecipitated chromatin was subjected to qRT-PCR. Data are presented as fold enrichment relative to IgG, with values normalized to *Gnrh1* and expressed as mean ± SEM. Statistical analysis by one-way ANOVA followed by a Tukey post-hoc, *p < 0.05, **p < 0.01, as indicated by bar. n.s.: non-significant. **d** LβT2 cells were treated with GnRH and/or activin (*n* = 3), before DNA from nuclei were treated with increasing concentrations of DNaseI and analyzed by qRT-PCR with primers specific to *Lhb*. Amplicon quantities were normalized to untreated samples (Vehicle), and data presented as the mean fraction of DNA remaining relative to vehicle

Opening of the chromatin, along with activating histone modifications, promotes access to the chromatin of transcription factors. It has previously been shown that the known gonadotrope regulatory proteins PITX2 (*Pitx2*), SF1 (*Nr5r1)*, and LHX3 (*Lhx3)* control expression of the mature gonadotrope markers *Lhb*, *Fshb*, and *Gnrhr* [25, 46, 47]. Using RNA sequencing, we show that these transcription factors are expressed in the gonadotrope lineage and thyrotrope cell lines, but not in NIH3T3 cells (Fig. 4a). Appropriately, the gonadotrope-specific transcription factor SF1 is not expressed in thyrotropes (TαT1) or in the gonadotrope progenitor cells (αT1–1). To test whether the gonadotrope-specific regulatory factors, LHX3 and PITX1, are associated with regulatory regions of gonadotrope promoters in any of the studied cell lines, we performed a ChIP analysis using LHX3 and PITX1 antibodies. ChIP analysis revealed that both transcription factors can bind on the gonadotrope terminal target genes (Fig. 4b, c). LHX3 is able to bind the promoter of *Cga* and *Gnrhr*, but neither *Lhb* nor *Fshb* exhibit binding by this method, at any of the stages of development (Fig. 4b, LHX3). PITX1 binds the gonadotrope-specific promoters *Cga* and *Lhb*, but not *Gnrhr* nor *Fshb* in this assay (Fig. 4c). This suggests the program of differentiation in these cells is not regulated simply by deacetylated histones [48], but more likely requires a timed balance of transcription factors, co-factors, histone modifications, DNA methylation, and/or networks to control the developmental gonadotrope gene expression program.

To extend this further, we asked if the final differentiation of the gonadotrope lineage cells required GnRH and/or activin treatment for the proper expression of mature gonadotrope genes. We serum starved LβT2 cells for 16 h then treated them for 4 h with 100 ng/ml GnRH ±25 ng/ml activin and assayed the chromatin status of the *Lhb* promoter for DNase sensitivity. Indeed, activin with or without GnRH treatment promoted *Lhb* promoter opening (Fig. 4d, Two-way ANOVA compared to vehicle, *p* > 0.05 for activin at 2 and 5 units of DNaseI, and *p* > 0.01 for activin + GnRH at 2 and 5 units of DNaseI, *p* < 0.05 when comparing activin to activin + GnRH). *Lhb* is known to be induced by either GnRH or activin [23]. In contrast, these treatments did not change chromatin opening of the already opened *Cga* and *Gnrhr* promoters and could not change the chromatin status of *Fshb* (data not shown).

## Discussion

**Fig. 5:**
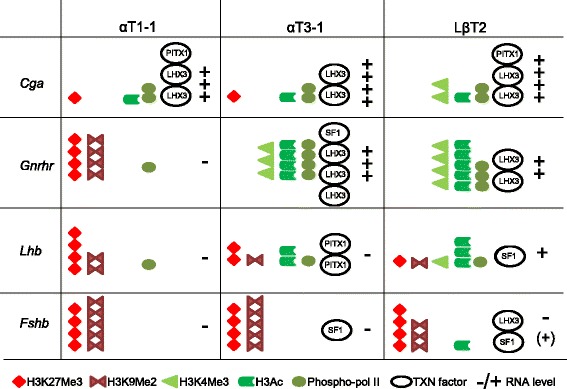
Summary of gonadotrope gene transcription, transcription factor occupancy and chromatin changes in gonadotrope cells. During gonadotrope maturation, illustrated here by the progenitor αT1–1, immature αT3–1, and mature LβT2 cells, the chromatin status changes from a closed conformation on *Gnrhr, Lhb* and *Fshb* (enrichment in H3K27Me3 and H3K9Me2) in αT1–1, to a more open conformation in LβT2 cells (increase in H3Ac, and H3K4Me3), which is associated with enhanced expression of *Lhb* and *Gnrhr* (+). The progressive opening of the *Lhb* promoter allows recruitment of the transcription factors (TXN factors) PITX1 and SF1 [68], whereas opening of the *Gnrhr* promoter allows recruitment of SF1 and LHX3 [69]. The regulatory region of the α-subunit *Cga* is in an open confirmation during all the stages of maturation, and associated with recruitment of PITX1 and LHX3 and high mRNA expression (+++). The discrete opening of the *Fshb* promoter during gonadotrope maturation allows association of SF1 and LHX3 to the regulator region of *Fshb* [70, 71]. Depending on study conditions, *Fshb* transcript is undetectable or can be detected at low levels in LβT2 cells

Elucidation of the molecular and cellular mechanisms underlying pituitary development and cellular specification is critical to our understanding of reproduction and infertility. Herein, we used three immortalized pituitary cell lines, which have been shown to model many aspects of developing gonadotropes [15, 20, 49]. It is clear that epigenetic regulation of chromatin is key in correct gene expression and cellular maturation. In this study, we present data showing that pituitary gonadotrope cell lines reveal epigenetic programming that allows the sequential expression of pituitary hormone genes in differentiation (Fig. 5).

The anterior pituitary has five different endocrine cell types: gonadotropes, lactotropes, corticotropes, thyrotropes, and somatotropes. Until now, no efficient method has been established to isolate live gonadotropes for in vitro cell culture. We have previously used the Ribotag technique, which allows selection of ribosome-associated mRNAs of specific cell-types, to isolate gonadotrope mRNAs and process them for qRT-PCR [50, 51]. However, as we were interested in comparing effects of hormone treatments and transcription factors on chromatin status, we performed this study in model gonadotrope cell lines. We used the developmentally staged gonadotrope cell lines, progenitor αT1–1, immature αT3–1, and mature LβT2 cell models to address the epigenetic regulation status of gonadotrope-specific genes (*Cga, Gnrhr, Fshb,* and *Lhb*) during gonadotrope maturation.

Studies of the chromatin state of *Cga* shows that it is open at a very early stage represented by αT1–1 cells, while *Gnrhr* chromatin opens at an immature developmental stage represented by αT3–1. These correlate directly with recruitment of phospho-Pol II and mRNA expression (Fig. 5). The *Lhb* and *Fshb* genes only begin to show relaxation of chromatin status in the more developmentally mature cell lines, αT3–1 and LβT2. This opening of the chromatin correlates with the appearance of positive, and loss of negative, histone modifications, which correlates with specific gene expression. We found that both *Gnrhr* and *Lhb*, though not *Fshb*, have progressive increases in phospho-Pol II, H3Ac and/or H3K4Me3, as well as a loss of H3K27Me3 and/or H3K9Me2 from the progenitor αT1–1 and immature αT3–1, to the mature LβT2 (Fig. 5). This partial correlation between chromatin state and gene transcript levels was recently described in αT1–1, αT3–1, and LβT2 cells, where Laverriere *et al.* were unable to detect *Fshb* and found the *Lhb* promoter to be equally sensitive to DNaseI treatment in αT1–1, αT3–1, and LβT2, despite transcription of *Lhb* specifically in LβT2 cells [5]. Interestingly, despite lack of *Fshb* transcription in the mouse fibroblast cell line, NIH3T3, our DNaseI sensitivity assay found the *Fshb* regulatory region to be relatively sensitive to DNaseI. DNaseI Chip-seq of NIH3T3 cells has been done previously and is available on the USCS genome browser (consulted July 25, 2017). The deposited data set was generated using a different DNaseI protocol from ours, using a single DNaseI concentration, and showed the *Fshb* and *Lhb* regulatory regions to be in relatively closed conformations. However, by comparing chromatin status and transcription factor occupancy of the *Fshb* regulatory region of numerous studies deposited on the USCS genome browser, we found the upstream region of *Fshb* to be able to bind transcription factors including REST (repressor element-1 silencing transcription factor), which is a known repressor of transcription [52]. Thus, it is very likely that the DNaseI sensitivity of the *Fshb* regulatory region, in NIH3T3 cells in our assay, shows some degree of opening. In addition, approximately 1 Kb upstream of the transcriptional start site of *Fshb* is a SINE repeat [53], which can allow expression in many different cell lines, and thus could confer a DNaseI sensitive region upstream of the *Fshb* gene. Interestingly, a SINE repeat has been found within intron 1 of the pig *Fshb* gene, which affects *Fshb* expression and reproductive function in pigs [54]. It will therefore be of interest in future studies to address the role of the SINE repeats upstream of the mouse *Fshb* transcription start site, to determine if these also impact *Fshb* expression in gonadotrope cell lines.

As expected, *Cga*, which is highly transcribed in all three cell lines, did not have significant differences between the cell lines in histone modifications or phospho-Pol II occupation. Although we and others [5] did not detect *Fshb* mRNA in any of the three studied cell lines without using highly sensitive qRT-PCR or activin treatment, *Fshb* does start to gain H3Ac histone modifications in LβT2 cells as well. Since *Fshb* is detected at low levels, and only using qRT-PCR in LβT2 cells [55], it is not unexpected that histone modifications may reflect a less active status (Fig. 5). We believe that the low expression of *Fshb* in LβT2 cells is due to the relatively simple milieu in which the cell lines are maintained, in comparison to the broad range of stimuli and hormones received by gonadotropes in vivo. The low expression of *Fshb* is possibly mediated by HDAC. It was previously shown that a GnRH treatment or inhibition of HDACs in αT3–1 cells allowed rapid expression of both *Lhb* and *Fshb* [56]. This confirms that αT3–1 cells possess all of the required transcriptional machinery to initiate expression of *Lhb* and *Fshb,* but removal of HDACs, particularly HDAC4 from these promoters is required to actively transcribe *Lhb* and *Fshb* [56]. Based on this, we hypothesized that hormonal treatment of LβT2 cells would allow opening of the chromatin of these genes. Thus, we treated LβT2 cells with activin ± GnRH. Indeed, activin significantly promoted *Lhb* promoter opening in LβT2 cells with or without GnRH, however we were unable to detect opening of the *Fshb* promoter in any of the studied conditions (not shown). As GnRH alone was inefficient in opening the chromatin of the *Lhb* promoter, perhaps due to the tonic as opposed to pulsatile treatment or the single time point of a 4-h exposure. GnRH does induce *Lhb* gene expression but may act without further opening the chromatin. Prior studies have shown that an increase in GnRH pulse frequency favors *Lhb* expression [57, 58], while a decrease favors *Fshb* gene expression [59–61]. To further our understanding of chromatin status of gonadotrope genes, and their response to hormone treatment, it will be of interest to expand the presented studies using Chip-seq, which allows the study of enhancers as well as other regulatory regions, in combination with ATAC-seq, a technique which has been shown to be more precise than H3K27Ac in identifying active promoters [62].

To establish the relationship between molecular and epigenetic mechanisms in gonadotropes, we investigated the accessibility of the transcription factors, LHX3, and PITX1, to bind gonadotrope-specific gene promoters in the studied cell lines [63–67]. Interestingly, the regulatory proteins PITX1/2, SF1, and LHX3 were all present in the intermediate gonadotrope cell line (αT3–1), and some even in αT1–1 (PITX1/2, and LHX3). By comparing chromatin status and transcription factor access to the chromatin, our data indicate that LHX3 is important in early and maintained expression of *Cga*. In agreement with previous studies [25, 31], the opening of the *Gnrhr* promoter in αT3–1 cells allowed increased association of LHX3 with the promoter, whereas the increased opening of the *Lhb* promoter correlated with recruitment of PITX1 (Fig. 5) [68]. In our hands, the *Fshb* promoter was in a rather closed configuration, not allowing detectable association of transcription factors with this gene.

## Conclusion

Our data show exciting new evidence of gonadotrope-specific chromatin changes taking place during development using immortalized model gonadotrope cell lines. Although the cell lines utilized remain an in vitro system, representative of developing gonadotrope cell lines, we show a progressive opening of the gonadotrope-specific gene promoters, *Cga*, *Gnrhr*, *Lhb*, and *Fshb* in these model cell lines and demonstrate that these promoters are not only controlled by developmental transcription factors, but also by epigenetic mechanisms that include the modulation of chromatin structure, and histone modifications.

## Abbreviations

Actb: Beta actin
Cga: Glycoprotein hormones or αGSU
ChIP: Chromatin immunoprecipitation
E: Embryonic day
FSH: Follicle-stimulating hormone
Fshb: FSH beta subunit
GnRH: Gonadotrope-releasing hormone
GnRHR: Gonadotropin-releasing hormone receptor
H3Ac: H3 acetylation
H3K4: Histone H3-lysine 4
H3K4Me3: H3K4 trimethylation
HDAC: Histone deacetylases
LH: Luteinizing hormone
Lhb: LH beta subunit
phospho-Pol II: phosphorylated polymerase II
SF1: Steroidogenic Factor 1 or Nr5a1
